# Comorbidities, intensity, frequency and duration of pain, daily functioning and health care seeking in local, regional, and widespread pain—a descriptive population-based survey (SwePain)

**DOI:** 10.1186/s12891-015-0631-1

**Published:** 2015-07-24

**Authors:** Anna Grimby-Ekman, Björn Gerdle, Jonas Björk, Britt Larsson

**Affiliations:** 1 Occupational and Environmental Medicine, University of Gothenburg, Gothenburg, Sweden; 2 Division of Community Medicine, Department of Medical and Health Sciences, Faculty of Health Sciences, Linköping University, Pain and Rehabilitation Center, Anaesthetics, Operations and Specialty Surgery Center, County Council of Östergötland, Linköping, Sweden; 3 Division of Occupational and Environmental Medicine, AMM, Lund University, Lund, Sweden

**Keywords:** Population-based, Local pain, Regional pain, Widespread pain, Comorbidities, Implications, Transition

## Abstract

**Background:**

The clinical knowledge of factors related to the spread of pain on the body has increased and understanding these factors is essential for effective pain treatment. This population-based study examines local (LP), regional (RP), and widespread pain (WSP) on the body regarding comorbidities, pain aspects, and impact of pain and elucidates how the spread of pain varies over time.

**Material and methods:**

A postal questionnaire that addressed pain aspects (intensity, frequency, duration and anatomical spreading on a body manikin), comorbidities and implications of pain (i.e., work situation, physical activity, consumption of health care and experience of hospitality and treatment of health care) was sent to 9000 adults living in southeastern Sweden. Of these, 4774 (53 %) completed and returned the questionnaire. After 9 weeks, a follow-up questionnaire was sent to the 2983 participants who reported pain in the first questionnaire (i.e. 62 % of 4774 subjects). Of these, 1940 completed and returned the questionnaire (i.e. 65 % of 2983 subjects). The follow-up questionnaire included the same items as the first questionnaire.

**Results:**

This study found differences in intensity, frequency and duration of pain, comorbidities, aspects of daily functioning and health care seeking in three pain categories based on spreading of pain: LP, RP and WSP. Compared to the participants with RP and LP, the participants with WSP had lower education and worse overall health, including more frequent heart disease and hypertension. In addition, participants with WSP had more intense, frequent, and long-standing pain, required more medical consultations, and experienced more impact on work. The participants with RP constituted an intermediate group regarding frequency and intensity of pain, and impact on work. The participants with LP were the least affected group regarding these factors. A substantial transition to RP had occurred by the 9-week follow-up.

**Conclusions:**

This study shows an association between increased spread of pain and prevalence of heart disease, hypertension, more severe pain characteristics (i.e., intensity, frequency and duration), problems with common daily activities and increased health care seeking. The WSP group was the most affected group and the LP group was the least affected group. Regarding these factors, RP was an obvious intermediate group. The transitions between the pain categories warrant research that broadly investigates factors that increase and decrease pain.

**Electronic supplementary material:**

The online version of this article (doi:10.1186/s12891-015-0631-1) contains supplementary material, which is available to authorized users.

## Background

Pain can be limited or cover more or less the entire body. In cohorts of patients with chronic pain it has been observed that such spreading can be associated with certain sociodemographic characteristics and aspects of lower health and quality of life. Hence, worse clinical pictures, including activity limitations and participation restrictions when widespread pain (WSP) was present, have been reported [1–4].

There are several population based studies investigating the prevalence of WSP and associated factors. One common way to indicate the spreading of pain is number of pain sites using a predefined manikin. For people with chronic low back pain (CLP), the extent of pain has recently been shown to be associated with low level of education, low social class, disability pension application, and clinical variables such as pain intensity and medical consultations [5]. A Norwegian population study reported that number of pain sites was linearly related to decreased function [6] and linked to future disability [7]. Identifying the number of painful sites as a way to assess the spreading of pain has some advantages in epidemiological studies, but this approach does not link population epidemiology to clinical medicine. This method of assessment does not take into consideration the anatomical distribution of the painful areas and may therefore have limited validity in the clinical context. Spatial categories frequently used in the clinical situation are instead local (LP), regional (RP) and widespread pain (WSP) [8, 9]. Epidemiological studies using such an approach have focussed upon WSP, which generally has been defined according to the American College of Rheumatology (ACR) using a manikin: pain in two contralateral quadrants and in the axial skeleton present for at least three months [10]. Most studies have investigated *chronic* widespread pain (CWP), which may be relevant since the majority of WSP (>90 %) in the population appear to be chronic [11]. In the Western population, the prevalence of CWP is approximately 5–15 % and the prevalence of self-reported chronic pain is approximately 50 % [12–16]. Peripheral factors (e.g., trigger points) and central nervous alterations probably contribute to the initiating and perpetuating of CWP [17]*.* Epidemiological studies usually compare subjects with no pain with subjects with CWP, these studies do not provide knowledge of the broad range of pain conditions present in populations (i.e., the majority of the pain conditions). One population study, however, reported that health status discriminated between subjects with no pain, pain not defined as CWP, and CWP [18]. Another study found that CWP had a greater impact than chronic neck pain with respect to pain duration and working capacity [19].

Some population studies of chronic pain have investigated psychological co-morbidities [20–22] and a few studies have investigated physical co-morbidities [23]. For subjects with CWP one study has reported an increased risk for hospitalization [24]. Increased risk of dying of cancer and cardiovascular diseases during a 2-year follow-up period in CWP have also been found [25]. A better understanding is needed about the relationship between the spreading of pain and occurrence of a broad spectrum of comorbidities.

Although CWP - both in cohorts of patients and in population cohorts - is associated with a variety of negative consequences of the pain, few population studies have investigated whether, for example, general health, health care seeking behaviour, or demanding physical activity vary with respect to the whole range of pain spreading on the body. Although pain frequency and intensity have important implications with respect to health care seeking [16, 26], these pain aspects are generally not elucidated in relation to the degree of spreading of pain.

Pain exhibits occasional, intermittent or constant symptoms and varies from barely perceptible to unbearable. Several clinical and population-based studies have reported that the spreading of pain varies over time [27]. Because knowledge about the spreading of pain and under what circumstances the spreading of pain increases, decreases, or remains unchanged is derived mostly from studies that were not specifically designed to examine these issues, definite conclusions are difficult to draw.

To this end, this study examines differences in sociodemographics and health aspects between individuals with different degrees of spreading of pain (defined as LP, RP, and WSP) and individuals who are pain-free to investigate whether spreading of pain is related to other pain aspects (frequency, intensity and duration) and implications of pain (i.e., work situation, physical activity, consumption of health care and experience of hospitality and treatment of health care).

The following research questions are addressed in this study, which is a report from a Swedish epidemiological project of pain in the community (SwePain):

- Are there differences regarding sociodemographics, comorbidities, general health, and physical activities between individuals with and without pain?
- Is the spreading of pain (defined as LP, RP, and WSP) associated with co-morbidities, limitation of professional work and daily chores, limitation of physical activity, increased health care seeking behaviours as well as pain intensity, frequency, and duration?
- What pattern of transition between the three pain categories (LP, RP, and WSP) can be seen over a short period?

## Methods

### Subjects and questionnaires

**Fig. 1:**
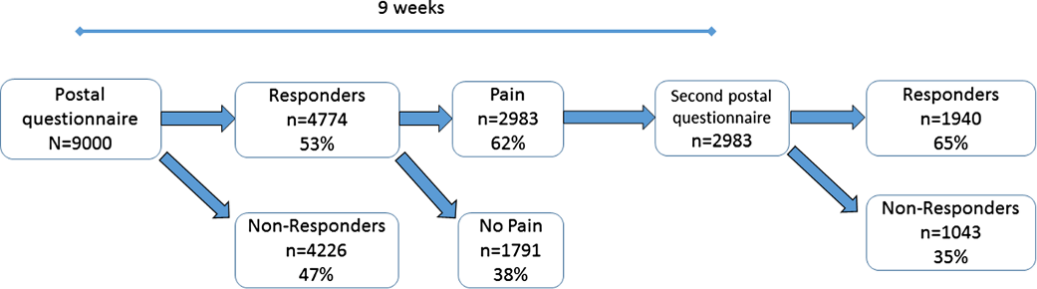
Flow chart showing study population: responders and non-responders to the first postal questionnaire and proportions with pain; and responders and non-responders to the second postal questionnaire at the 9-week follow-up

The subjects were selected from a sampling frame based on the Swedish Total Population Register. The sample frame consisted of 404 661 individuals living in southeastern Sweden. Using this sample frame, we collected a stratified simple random sample of 9000 people (16–85 years old) and then mailed these people a postal questionnaire (Fig. 1). The sampling frame was stratified according to municipality to reach subjects living in urban and rural areas, to ensure appropriate gender distribution, and to include sick leave status. Data were collected by Statistics Sweden. As partial missing is present in the study, the actual number of observations is reported for each analysis in the tables. Sick leave was defined as missing work for more than 45 consecutive days during 2009. In Sweden, musculoskeletal complaints/disorders are the second most common causes of sick leave. By stratifying the sample frame for all subjects on sick leave, we intended to select enough individuals with pain so future studies could use this data. The first questionnaire was returned either by post or electronically by 4774 subjects (53 %) and included 21 items that asked respondents about the following information: educational level (9-year compulsory school, upper secondary school, university education); doctor-diagnosed diseases—heart disease, hypertension, stroke, eczema, and pulmonary disease (yes or no); and perceived general health (five-grade Likert scale, from 1 (excellent) to 5 (poor)). All subjects who reported pain over the previous 7 days were also asked to answer questions about certain pain aspects: a) pain frequency during the previous week (using a four-grade Likert scale from 1 (always/nearly always) to 4 (seldom)), b) pain duration (less than 3 months or more than 3 months) and c) pain intensity the previous 7 days (eleven-grade Numeric Rating Scale (NRS) from not at all to worst imaginable pain).

In addition, these subjects were asked about implications of their pain: 1) influence of pain on professional work and daily chores (a five-grade Likert scale from 1 (not at all) to 5 (very much)); 2) influence of pain on demanding physical activities per week (a five-grade scale: 5 h or more; more than 3 h but less than 5 h; 1 to 3 h; and 1 h or a not at all (demanding physical activity was exemplified with activities such as running, jogging, tennis, squash, hard bicycling etc.); 3) consultation of complementary medicine related to pain during the previous 12 months (yes or no); 4) health care consultations related to pain during the previous 12 months (yes or no); and 5) experience of hospitality and treatment in health care (NRS from 0 (bad) to 10 (very good)). Subjects reporting pain were asked to mark the location of their pain on an anatomical sketch of a human being (i.e. a body manikin; see Additional file 1).

For those subjects who reported pain at any time during the previous 7 days (2983), a second questionnaire was mailed 9 weeks after the first one. Of these, 1940 (65 %) completed and returned the questionnaire. This second questionnaire included the same items found on the first questionnaire (sociodemographics, consequences of pain, and certain pain aspects) and an identical body manikin; from the second questionnaire we only report on the spreading of pain shaded on the body manikin.

For both questionnaires, a reminder was sent to non-responders after 2 weeks and, if necessary, sent after another 2 weeks.

The project was approved by the local ethics committee of Linköping University, Sweden, diary 2011 72/31.

### The three pain categories

The subjects marked the site of their pain on a body manikin divided into 22 sections on the front and 22 sections on the back. Using a slightly modified version of the Manchester definition of widespread pain [12, 28], we defined widespread pain (WSP) as pain in at least two sections in two contralateral limbs and the axial skeleton and marked equally on the front and on the back of the body. MacFarlane et al. defined widespread pain in limbs to be present “if there are at least two painful sections (in two contralateral limbs)” [28], a definition that does not require pain to be marked equally on the front and back of the body. Therefore, our study uses a more rigorous definition of widespread pain. When marked on just one section (or two sections when sections were equally marked on the front and back of the manikin, e.g., hip, knee, shoulder, or arm), pain was defined as local pain (LP). We defined regional pain (RP) as pain shaded on the manikin that did not meet the criteria for WSP or LP. For manikins illustrating the three pain categories see Additional file 1. In addition and to allow for comparisons with previous research, we also defined WSP according to ACR criteria (denoted WSP-ACR) [10], so pain was considered widespread when present in both the left and right side of the body and also above and below the waist. In addition, our definition of WSP-ACR required the presence axial skeletal pain (i.e., in the cervical spine, the anterior chest, the thoracic spine, or the lower back).

We intended to register all types of pain conditions. According to other similar epidemiological studies most pain conditions will be considered as musculoskeletal pain conditions [29]. However, a confident differentiation between pain conditions is not possible with an epidemiological approach.

### Categorization of items on education level, general health, pain frequency, consequnecs for work and physical activities

Five items were dichotomized in order to discern negative implications of pain from insignificant implications of pain: 1) The item educational level was dichotomized to *low* when reported as 9-year compulsory school or upper secondary school levels and to *high* when reported as university education. 2) General health was dichotomized to *decreased* when general health was reported “poor” or “fairly poor” and to *not decreased* when general health was reported “good”, “very good”, or “excellent”. 3) Pain frequency was dichotomized to *frequent* when pain was reported to be present “very often” or “always” and to *not frequent* when pain was reported to be present “sometimes” or “seldom”. 4) Consequences for professional work and daily chores was dichotomized to *present* when ability to work professionally was considered to be “much” or “very much” influenced by pain and *not present* when ability to work was considered “not at all” or “moderately” influenced by pain. 5) Demanding physical activities per week was dichotomized to *maximally 1 h per week* and *more than 1 h per week*.

#### Statistical analysis

All data were analysed using the Statistical Package for Social Sciences (SPSS) version 20. Level of statistical significance was set at *P* < 0.01. The differences of proportions of comorbidities between subjects with and without pain were calculated together with 95 % confidence intervals (CI) [30]. All pair-wise comparisons of proportions were based on the following calculation of a z-statistic:

$$ \mathrm{z}=\frac{{\mathrm{p}}_1-{\mathrm{p}}_2}{\mathrm{SE}} $$

$$ \mathrm{S}\mathrm{E}=\sqrt{\mathrm{p}\ast \left(1\hbox{-} \mathrm{p}\right)\ast \left[\left(1/{\mathrm{n}}_1\right)+\left(1/{\mathrm{n}}_2\right)\right]} $$

$$ \mathrm{p}=\raisebox{1ex}{$\left(\left[{\mathrm{p}}_1\ast {\mathrm{n}}_1\right]+\left[{\mathrm{p}}_2\ast {\mathrm{n}}_2\right]\right)$}\!\left/ \!\raisebox{-1ex}{$\left({\mathrm{n}}_1+{\mathrm{n}}_2\right)$}\right. $$

The approximate p-values were then retrieved from a normal distribution table. The pair-wise comparisons of pain intensity as well hospitality and treatment from health care service were made using the Mann Whitney U-test. Comparisons between mean-age in different groups were made using the independent samples T-test. The Wilson method was used to calculate 95 % CIs for the baseline and the 9-week follow-up proportions of the different pain categories [30]. In these calculations, the proportions are weighted to better reflect the general population (see below) and to keep the sample size of the study unchanged.

### Weighted data

The prevalence and proportions presented are based on weighted data. Weighting the data adjusts for miss-representation due to design and non-response. This adjustment means the data better represent the population being studied. To calculate this adjustment, a weight is calculated for each individual:

$$ \mathbf{w}\mathbf{k}=\mathrm{d}\mathbf{k}\ast \mathbf{v}\mathbf{k} $$

where **wk =** weight for individual k, d**k =** adjustment due to the design, and **vk =** adjustment due to non-response.

The value for each individual was then multiplied by the individual weight. The statistical entities calculated using the weighted observations more accurately represent the whole population than statistics calculated using un-weighted observations. Weighting the data corrects for some of the biases inherent in the design and as a result of non-uniform non-response. For a thorough description of estimation of weights, see Lundström and Särndal [31].

The weights were calculated based on the following auxiliary variables: gender, municipality affiliation, registered sick-leave in 2009 (more than 45 consecutive days), age, marital status, educational level, and country of birth. The distribution of these auxiliary variables in the original sample as well as in the weighted sample is shown in Table 1.

**Table 1:** Description of background variables for non-weighted and weighted samples

Auxiliary variable		Non-weighted sample	Weighted sample
	N	Years	Years
Age			
25^th^ percentile	4744	41	29
50^th^ percentile		54	45
75^th^ percentile		64	62
	N	%	%
Gender			
Men	2148	45	50
Women	2626	55	50
Country of birth			
Sweden	4191	88	84
EU 27 + Scandinavia (except Sweden)	192	4	4
Other countries	391	8	12
Educational level			
9-year compulsory school	1250	26	24
Upper secondary school	1988	42	43
University education	1482	31	33
Missing	54	1	1
Marital status			
Married	2467	52	42
Unmarried	2307	48	58
Municipality affiliation			
Urban area	4125	86	93
Rural area	649	14	7
Registered sick-leave more than 45 consecutive days in 2009	2134	45	1

EU 27+ Scandinavia = the 27 countries of the European Union (i.e., except Sweden) and Norway and Iceland

## Results

### Response to postal questionnaire and prevalences of the three pain categories

Of the 4371 respondents from the first questionnaire, 66 % (2880) reported pain and 1491 reported no pain during the previous 7 days. Mean age in the pain group was 52 years (standard deviation (sd) 15 years) and mean age in the no-pain group was 51 years (sd 17 years) (*p*-value < 0.001). No significant difference in marital staus existed (pain group: 51.9 % married vs. non-pain group: 51.5 %; *p* = 0.785). The proportion of women was significantly higher in the pain group than in the non-pain group (59.5 % vs. 47.1 %; *p* < 0.001).

In total, 103 subjects were excluded from the analyses as they reported pain the previous 7 days but in the first questionnaire they did not mark on the manikin; corresponding figure for the second questionnaire was 26 subjects. Of the 4371 respondents, 10 % (*n* = 418) reported LP, 51 % (*n* = 2216) RP, and 6 % (*n* = 246) WSP. Corresponding proportions in the pain group were 15 % LP, 77 % RP, and 9 % WSP. Using the ACR criteria, we concluded that 10 % of the total respondents had WSP-ACR (*n* = 418) and 52 % (*n* = 2482) had reported pain not defined as widespread.

The three pain categories (LP, RP and WSP) were quite distinct when it comes to the number of pain sites out of 45 anatomical regions on the body manikin. All responders with LP had only 1 to 2 pain sites, by definition, and with median of 1 pain site. The responders in RP reported a median number of pain sites of 4, and the 10th and 90th percentile were 2 to 10 respectively. For the responders in WSP the median number of pain sites were 25, and the 10th and 90th percentile were 11 to 40 respectively.

For those 2983 subjects who reported pain at any time during the previous 7 days in the first questionnaire, a second postal was mailed 9 weeks after the first one (Fig. 1). Of these, 1940 (65 %) completed and returned the questionnaire.

### Pain free subjects versus subjects with pain

The prevalences of the all registred comorbidities (except stroke) were statistically significantly higher for the subjects with pain than for the subjects without pain (Table 2). This difference was also the case for decreased general health, reduced time spent on physically demanding daily activities, and low educational level.

**Table 2:** Weighted prevalence in per cent (%) of comorbidities, general health, physical activity, and low educational level for individuals reporting pain and those not reporting pain

n_p_ = 2983, n_np_ = 1491	Pain %	No pain %	Difference %	95 % CI	*P*-values
Heart disease	11.0	6.7	4.3	2.53; 6.08	<0.001*
n_P_ =2466, n_np_ =1387					
Hypertension	26.5	18.7	7.8	5.20; 10.34	<0.001*
n_p_ = 2715, n_np_ = 1488					
Stroke	3.1	1.9	1.1	0.6; 1.7	0.019
n_p_=2385, n_np_ = 1470					
Diabetes	7.3	5.4	1.9	0.35; 3.45	<0.001*
n_p_ = 2441, n_np_ = 1474					
Eczema	22.9	14.4	8.5	6.06; 1.7	<0.001*
n_p_ = 2492, n_np_ = 1489					
Pulmonary disease	7.3	4.1	3.2	1.77; 4.63	<0.001*
n_p_ = 2442, n_np_ = 1460					
Decreased general health	27.6	6.1	21.5	19.5; 23.5	<0.001*
n_p_ = 2963, n_np_ = 1407					
Maximally 1 h weekly spent on demanding physical activities	41.7	34.5	7.2	5.8; 7.6	0.001*
n_p_ = 2967, n_np_ = 1409					
Low educational level	72.2	62.1	10.1	7.28; 12.83	<0.001*
n_P_ = 2963, n_np_ = 1407					

The far right indicates differences between the two groups, 95 % confidence interval (CI), and p-values

*n*_*p*_ response rates in pain group, *n*_*np*_ response rates in no-pain group

*Denotes significant group difference

### Social and educational levels in the three pain categories

Statistically, the proportion of women was significantly highest in WSP, lowest in LP, and intermediate in RP (Table 3). Married subjects comprised nearly half the pain categories and were not statistically significantly different between the three pain categories. The proportions of subjects with low education were statistically significantly higher in WSP than in RP and LP. The proportions of low education in the latter categories were not statistically significantly different.

**Table 3:** Demographic variables in the three pain categories based on spreading of pain presented as weighted prevalence (%). Statistical comparisons are furthest to the right^a^

Pain category Variables	Local pain (*n* = 414–418)	Regional pain (*n* = 2200–2203)	Widespread pain (*n* = 244–245)	Statistics (*p*-value)
Women (%)	51.4	59.4	75.6	LP vs. RP: <0.001*
				LP vs. WSP: <0.001*
				RP vs. WSP: <0.001*
Married (%)	50.2	52.0	52.8	LP vs. RP: 0.490
				LP vs. WSP: 0.818
				RP vs. WSP: 0.320
Low education level (%)	69.6	70.8	82.4	LP vs. RP: 0.740
				LP vs. WSP: <0.001*
				RP vs. WSP: < 0.001*
Mean age (years (sd))	53.3 (16.1)	51.7 (15.0)	52.2 (11.2)	LP vs. RP: 0.005*
				LP vs. WSP: 0.135
				RP vs. WSP: 0.161

Range of response rate (n) across the variables is in the pain category column

*LP* local pain, *RP* regional pain, *WSP* widespread pain

*Denotes statistical group difference

^a^Differences in proportions are tested pair-wise

### Comorbidities and general health in the three pain categories

Proportions of subjects with heart disease, hypertension, diabetes and decreased general health were statistically significantly higher in WSP than in LP (except for hypertension) and in RP (Table 4). The proportions of subjects with heart disease, hypertension, or decreased general health were not statistically significantly different between LP and RP. The proportions of subjects with stroke, pulmonary disease or eczema were not statistically significantly different between the three pain categories.

**Table 4:** Weighted proportions of co-morbidities in per cent for the three pain categories based on spreading of pain. Statistical comparisons are furthest to the right^a^

Pain category Variables	Local pain (*n* = 330–417)	Regional pain (*n* = 1785–2203)	Widespread pain (*n* = 199–244)	Statistics (*p*-value)
Heart disease (%)	9.5	10.0	20.5	LP vs. RP: 0.770
				LP vs. WSP: 0.001*
				RP vs. WSP: <0.001*
Hypertension (%)	24.9	25.2	34.3	LP vs. RP: 0.617
				LP vs. WSP: 0.013
				RP vs. WSP: <0.001*
Stroke (%)	3.3	2.9	3.0	LP vs. RP: 0.940
				LP vs. WSP: 0.850
				RP vs. WSP: 0.513
Eczema (%)	5.8	7.3	5.6	LP vs. RP: 0.022
				LP vs. WSP: 0.920
				RP vs. WSP: 0.358
Diabetes (%)	18.1	23.7	37.0	LP vs. RP: 0.329
				LP vs. WSP: <0.001*
				RP vs. WSP: 0.001*
Pulmonary disease (%)	8.3	6.8	8.4	LP vs. RP: 0.320
				LP vs. WSP: 0.960
				RP vs. WSP: 0.392
Decreased general health (%)	14.4	27.2	70.3	LP vs. RP: 0.051
				LP vs. WSP: <0.001*
				RP vs. WSP: <0.001*

Range of response rate (n) across the variables is in the pain category column

*LP* local pain, *RP* regional pain, *WSP* widespread pain

*Denotes statistical group difference

^a^Differences in proportions are tested pair-wise

### Frequency, duration and initensity of pain in the three pain categories

The proportions of subjects with frequent pain (very often or always) was statistically significantly highest in WSP, statistically significantly lowest in LP, and statistically significantly intermediate in RP (Table 5). The proportion of subjects with pain more than 3 months was statistically significantly highest in WSP and not statistically different between LP and RP. The pain intensity differed significantly between the three groups and was lowest in LP and highest in WSP.

**Table 5:** Weighted proportions in per cent (%) of different pain characteristics and median pain intensity in the three pain categories based on spreading of pain on the body. Statistical comparisons are furthest to the right^a^

Pain category Variables	Local pain (*n* = 403–416)	Regional pain (*n* = 2106–2173)	Widespread pain (*n* = 241–244)	Statistics (*p*-value)
Pain very often or always (%)	51.1	63.3	99.9	LP vs. RP: <0.001*
				LP vs. WSP: <0.001*
				RP vs. WSP: <0.001*
Pain duration > 3 months (%)	60.1	68.6	92.4	LP vs. RP: 0.130
				LP vs. WSP: <0.001*
				RP vs. WSP: <0.001*
Pain intensity last 7 days (median; (1^st^ and 3^rd^ quartile))^b^	4 (3, 6)	5 (3, 6)	7 (6,8)	LP vs. RP: <0.001*
				LP vs. WSP: <0.001*
				RP vs. WSP: <0.001*

Range of response rate (n) across the variables is in the pain category column

*LP* local pain, *RP* regional pain, *WSP* widespread pain

*Denotes statistical group difference

^a^Differences in proportions are tested pair-wise

^b^The mean pain intensity, and sd in parantheses, were in each pain group as follows: 4.2 (1.9), 4.9 (1.9) and 6.5 (1.8)

### Daily functioning and health care seeking in the three pain categories

The proportion of great influence on work and daily chores was highest in WSP, lowest in LP, and intermediate in RP (Table 6). The proportion of subjects who had sought health care service the previous 12 months was statistically significantly highest in WSP; the other categories were not statistically different. Experience of hospitality and good treatment from health care was statistically significantly lower in WSP than in LP with RP intermediate. The proportions of subjects who had sought complementary health care during the previous year were not statistically different between the categories. Proportions of decreased time participating in physically demanding activities (maximally 1 h per week) was statistically significantly lower in LP than in WSP.

**Table 6:** Weighted proportions in per cent (%) of different implications of pain in the three pain categories based spreading of pain on the body. Statistical comparisons are furthest to the right^a^

Pain category Variables	Local pain (*n* = 400–417)	Regional pain (*n* = 2261–2205)	Widespread pain (*n* = 242–245)	Statistics (*p*-value)
Maximally 1 h weekly spent on demanding physical activities (%)	36.4	42.3	46.5	LP vs. RP: 0.025
				LP vs. WSP: 0.001*
				RP vs. WSP: 0.321
Impact on ability to work or to perform daily chores (%)	12.6	19.0	74.6	LP vs. RP: 0.002*
				LP vs. WSP: <0.001*
				RP vs. WSP: <0.001*
Complementary health care last 12 months (%)	19.5	19.4	21.7	LP vs. RP: 0.980
				LP vs. WSP: 0.500
				RP vs. WSP: 0.390
Health care previous 12 months (%)	37.1	40.5	81.5	LP vs. RP: 0.260
				LP vs. WSP: <0.001*
				RP vs. WSP: <0.001*
Hospitality and treatment from health care service (%)	8 (0–10)	7 (0–10)	6 (0–10)	LP vs. RP: 0.018
				LP vs. WSP: <0.001*
				RP vs. WSP: 0.028

Range of response rate (n) across the variables is in the pain category column

*LP* local pain, *RP* regional pain, *WSP* widespread pain

*Denotes statistical group difference

^a^Differences in proportions are tested pair-wise

### Transition between the three pain categories at 9-week follow-up

At the 9-week follow-up, 64 % of the subjects with WSP remained in the same category and 36 % of the WSP subjects had transitioned to the RP category (Table 7). For LP, 44 % had transitioned into RP and none into WSP. For RP and LP, the proportions remaining in the same pain category were 88 and 56 %, respectively. For RP, 3 % had transitioned into WSP and 10 % into LP. For LP 44 % had transitioned into RP and none to WSP. At the follow-up, 13 % of LP, 7 % of RP, and 1 % of WSP reported no pain the previous 7 days.

**Table 7:** Weighted prevalence in per cent (%) of transition from pain categories at baseline to 9-week follow-up

After 9 weeks →	Local pain	Regional pain	Wide spread pain	Column total	No pain previous 7 days
At baseline↓	% (95 % CI)	% (95 % CI)	% (95 % CI)	%	% (95 % CI)
Local pain *n* = 184	56 (48.5; 62.7)	44 (37.3; 51.5)	0 (0.0; 2.0)	100	13 (9.2; 18.5)
Regional pain *n* = 1205	10 (8.2; 11.6)	88 (85.5; 89.2)	3 (1.9; 3.8)	100	7 (5.7; 8.5)
Widespread pain *n* = 146	0 (0.0; 2.6)	36 (28.9; 44.4)	64 (55.6; 71.1)	100	1 (0.2; 4.1)

*95 % CI* 95 % confidence interval

## Discussion

The most important results of this study are listed below.

- Subjects with pain had lower educational levels, more comorbidities, decreased general health, and decreased physical activities than the pain-free subjects.
- Differences in comorbidities, certain pain aspects, daily functioning and health care seeking in the three pain categories based on spreading of pain were found.
- Low education, heart disease, hypertension, diabetes, decreased general health, increased medical consultation, high impact on work, and intense, frequent, and chronic pain were more frequent in WSP than in RP and LP.
- Regarding impact of work and frequency and intensity of pain, RP was the intermediate group and LP was the least affected group.
- There was no difference between LP and RP regarding education, general health, duration of pain, and health care consumption.
- The proportion of women differed between the three pain categories; it was highest in WSP and lowest in LP. This difference was also the case for experience of hospitality and good treatment from health care: WSP rated this lowest and LP highest.
- In the pain categories, proportions of married subjects, physical demanding activities, and complementary health care did not differ.
- For both LP and WSP, a substantial transition to RP had occurred by the 9-week follow-up.

The higher prevalence of comorbidities and the pain itself might contribute to reports of decreased health, on average, among participants with pain. The limited time spent weekly on demanding physical activity among individuals with pain (Table 2) may indicate that many individuals answering “yes” for pain present during the previous 7 days have pain severe enough to affect their daily life, a finding in line with previous research on subjects with pain [18, 32, 33]. Heart disease and hypertension were among the diseases that differed most between individuals with and without pain (Table 2), a finding that coincides with previous epidemiologic research showing higher prevalence of hypertension in subjects with pain [34, 35]. Several mechanisms of interaction between cardiovascular and pain regulatory systems and possible alterations in homeostatic feedback to restore elevation of blood pressure in pain have been suggested [36], although the literature is contradictory [35, 37, 38].

Because more than 90 % of WSP had chronic pain (pain for more than 3 months), our use of WSP is very similar to the way previous research has used CWP. In our study, the prevalence of WSP was 6 %, a percentage similar to what a British population study found (5 %), which used the Manchester definition for CWP [12]. The use of the Manchester definition of CWP correlates to a population prevalence of CWP lower than the use of the ACR criteria. Studies that defined CWP according to ACR criteria [13, 39–41] found CWP prevalences of 11 %, 11 %, 15 %, and 12 %, respectively. Our study, when using ACR criteria to define WSP , found a slightly lower CWP prevalence (9 %) than these studies.

To the extent that heart disease in subjects reporting pain was equivalent to cardiovascular diseases (CVD), the findings coincide with earlier associations between CVD [25, 42] and chronic pain. Heart diseases and hypertension were reported to be most frequent in WSP (Table 4), a finding previously reported in CWP [25]. Painful diseases common in the general population, such as arthritis and osteoporotic fractures, are associated with elevated levels of CVD [43–47]. The overall occurrence of these diseases in the population might contribute to higher prevalence of CVD among individuals with pain. With respect to CVD [48] and to some extent chronic pain [49–52], there is support for pathogenic immune and inflammatory processes, but comorbidities may be independently linked to chronic pain [53]. Pain groups tend to have lower levels of education [39, 54–56], a finding that may reflect the complexity of factors interacting with pain. Low education may indicate working situations associated with higher risks for developing pain conditions [57, 58]. Individuals with less education tend to use less effective pain approaches [59]. That is, higher education might indicate better critical thinking skills that could help people make better health decisions and have more productive interactions with health care providers, all leading to better agency over one’s health [60]. The proportion of low educational level was highest in WSP (Table 3), a finding in line with previous studies [54, 61] but in contrast to a study on CWP and local pain—that study found no significant difference in educational level between the groups [2].

Subjects with WSP also reported higher health care use related to pain in the last 12 months than subjects with RP or LP (Table 6). Similarly, another study found that patients with widespread pain used primary health care services more often [62, 63]. On the other hand, no increased use of medical services has reported in subjects with CWP compared to subjects with more localised pain (i.e. CLP) [2].

In our study, subjects with WSP - compared to subjects with LP and RP - reported pain as more persistent and more frequent (Table 5). Similarly, Viniol and co-workers found these pain aspects to be more common in CWP than in subjects with CLP [2]. Subjects with WSP also reported significantly higher pain intensity than the two other pain categories (Table 5), findings that agree with studies reporting significant inter-correlations between spreading of pain and pain intensity [64, 65]. Furthermore, WSP was associated with the highest proportion of females (Table 3) and the highest proportion of subjects with decreased ability to work (Table 6), findings also reported in previous studies [2, 61]. In our study, a large majority of subjects with WSP also reported decreased general health (Table 4), a finding that is supported by several population-based studies [18, 66, 67]. Similarly, a case control study [68] reported significantly impaired overall health in subjects with CWP. The experience of hospitality and good treatment from medical care was lowest in the WSP group, at least partially reasonably reflecting a lack of satisfaction due to sparse effective treatment of WSP and the burden of the individuals to deal with extensive negative implications of pain. At the 9-week follow-up, none of subjects with previous WSP reported being pain free (Table 7).

Spreading of pain has been linearly correlated to impaired function [69], pain duration, and pain severity, which are all very common in WSP. In a systematic review [70], these symptoms were associated with poor outcome of pain. In our study, two-thirds of the WSP subjects remained in the category at the follow-up. A previous study found that only one-third of CWP remained in the category at follow-up [66]. These findings indicate possible improvement or natural fluctuations in the pain condition for individuals in this highly burdensome pain condition. The difference (two-thirds versus one-third) in improving from widespread pain (WSP and CWP, respectively) could be related to our study’s more rigorous definition of WSP. It is obviously not due to the fact that not all subjects in the present WSP group had chronic pain since >92 % had chronic widespread pain (Table 5). The least burdensome pain was found in the LP group. At follow-up, however, nearly half of LP had moved to the RP category, a worse situation with respect to intensity and frequency of pain and influence of pain on work. After 9 weeks, the majority of the RP group remained in the same category and 3 % of the RP group had moved to WSP. A systematic review [27], however, found no convincing link between follow-up time and the proportion of subjects transitioning from RP to CWP.

The use of LP and RP in this study needs some further explanation. Although previous literature has used the terms local and regional pain, our study uses the terms in a slightly modified way. Previous epidemiological studies have used both regional and local pain to describe broad groups defined as having less spreading of pain than WSP. In our study, LP is a clear and well-defined group consisting of individuals with pain at single locations and regional pain is a broad group that is defined to be in between LP and WSP regarding spreading of pain. To a large extent, in our study RP represented an intermediate group between LP and WSP regarding pain aspects and consequences of pain. In future studies, it would be interesting to split the broad group of regional pain into subgroups of increasing spreading. Sub-grouping also has to pay attention to that a minority of subjects with RP can have a higher number of pain sites than some of the subjects with WSP, which is due to the definitions of WSP requiring both a certain number of pain areas and a certain distribution on the body of these pain areas. However, we consider it important that such subgroups of RP have face-validity in a clinical perspective. If the results then still hold and show clear differences between all the pain groups, this would strengthen our hypotheses of a close to continuous characteristic of pain described by increasing spreading.

We define pain as a subjective experience according to the International Association for the Study of Pain: pain is an unpleasant and emotional experience [71]. Thus, the pain categories—i.e., self-reported pain marked on a manikin—also reflect subjective experiences. Our approach means that natural fluctuations in the spreading of pain per se - e.g. due to neurobiological, psychological and social factors - are possibly well captured in the transition between pain categories over the 9-weeks between the first and second questionnaire. Undoubtedly, in our study a small change in pain spreading can imply a movement between pain categories, although this movement is only applicable for individuals close to a boundary of a pain category. From a clinical point of view, because it is most probable to see small changes in the spreading of pain over nine weeks, it is not surprising that the main movements in pain categories were from LP to RP and from WSP to RP. However, it cannot be excluded that also test-retest reliability of the pain manikin may influence the transitions between the three pain categories. A part of the reliability aspect may be that the subject forget areas with intermittent or less interfering pain and instead focus upon the areas with most intense pain intensity when reporting in the questionnaire. In order to better understand the natural fluctuations in spreading of pain more frequent registrations, e.g. every day or several times per week, can be used. In the clinical situation most patients with long standing problems are repeatedly assessed in order to make a clinical characterization of the pain condition.

A strength of our study is the use of weights calculated by Statistics Sweden, as this approach improves the representativeness of the estimated prevalence and comparisons. However, the strata for sick-leave means we used an approximately equal proportion of subjects with and without sick-leave for more than 45 consecutive days, and it is likely that the proportions in the general population are different. Therefore, estimated proportions of comorbidities are more precise than estimations of the secondary consequences and pain aspects associated with sick leave.

There are some limitations of the present epidemiological study. Due to the nature of data collection it is not possible to investigate specific pain conditions in relation to non-specific pain conditions. Unfortunately, there are no valid algorithms that can be used for identifying a broad spectrum of specific pain conditions (diagnoses) in postal questionnaires used within the field of epidemiology. Clinical examinations are needed for diagnosing specific pain conditions.

A methodological issue important to note is the large amount of pairwise comparisons included in the study, therefore a significance level of 0.01 was chosen rather than 0.05. This lower p-value was chosen as some inferential conclusions are drawn, even if the main aim of the study is of a more descriptive nature. The issue of multiple testing might therefore not be a major concern both due to the descriptive nature of the study, and due to the fact that many of the p-values appear in nice patterns, not randomly.

## Conclusions

This study shows that there is an association between increased spreading of pain and prevalence of heart disease, hypertension, more severe pain characteristics (i.e., intensity, frequency and duration), problems with common daily activities and increased health care seeking. The most affected group was the WSP, the least affected group was the LP, and to considerable extent the intermediate group was RP. The transitions between the pain categories warrant future research that broadly investigates factors including reliabilty aspects that increase and decrease pain in the short- and long-term perspectives.

When clinically examining the patient with widespread pain it is important to pay attention to comorbidities in order to adequately plan treatments and interventions.

## Additional file

Additional file 1:
**It is a pdf file with manikins illustrating the three pain categories.**
