# Supplementary material for: Comorbidities, intensity, frequency and duration of pain, daily functioning and health care seeking in local, regional, and widespread pain—a descriptive population-based survey (SwePain)
Source: BMC Musculoskelet Disord. 2015 Jul 24;16:165. doi: 10.1186/s12891-015-0631-1 (PMC4511999; doi:10.1186/s12891-015-0631-1)

Additional file 1: Manikins illustrating the three pain categories. A) an empty manikin with 45 regions, B) an example of the local pain category (LP; two regions with pain), C) an example of the regional pain category (RP; five regions with pain) and D) an example of the widespread pain category (WSP; 21 regions with pain). Areas with pain are in gray color.

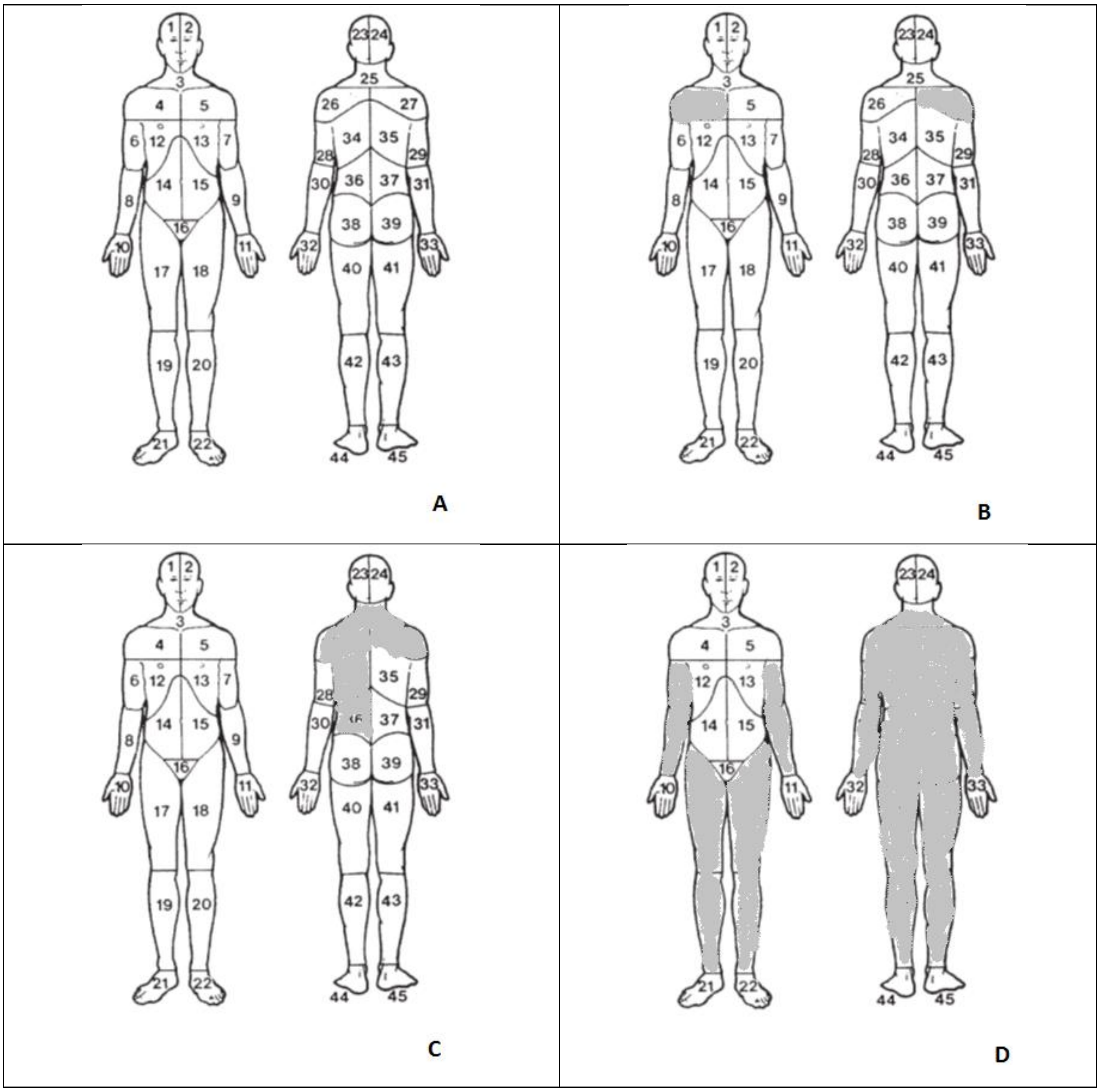

Supplement: Additional file 1: — It is a pdf file with manikins illustrating the three pain categories. [file 12891_2015_631_MOESM1_ESM.pdf]
